# Slow dynamics in features of synchronized neural network responses

**DOI:** 10.3389/fncom.2015.00040

**Published:** 2015-04-14

**Authors:** Netta Haroush, Shimon Marom

**Affiliations:** ^1^Department of Physiology, Faculty of Medicine, Technion—Israel Institute of TechnologyHaifa, Israel; ^2^Network Biology Research Laboratories, Faculty of Electrical Engineering, Technion—Israel Institute of TechnologyHaifa, Israel

**Keywords:** response variability, neural networks, excitation-inhibition balance, excitation-inhibition interaction, electrical stimulation, bicuculline, APV

## Abstract

In this report trial-to-trial variations in the synchronized responses of neural networks are explored over time scales of minutes, in *ex-vivo* large scale cortical networks. We show that sub-second measures of the individual synchronous response, namely—its latency and decay duration, are related to minutes-scale network response dynamics. Network responsiveness is reflected as residency in, or shifting amongst, areas of the latency-decay plane. The different sensitivities of latency and decay durations to synaptic blockers imply that these two measures reflect aspects of inhibitory and excitatory activities. Taken together, the data suggest that trial-to-trial variations in the synchronized responses of neural networks might be related to effective excitation-inhibition ratio being a dynamic variable over time scales of minutes.

## 1. Introduction

Evoked transient synchronous activity is acknowledged as significant in both normal and pathological neural conditions (Uhlhaas et al., 2009). In mammalian brains, as well as in their *ex-vivo* reduced experimental preparations (slices, cultured networks), an evoked transient synchronous activity has a temporally-stretched spike like shape in the population firing rate trace, a characteristic time scale (ca. 100 ms), the flavor of a threshold-governed event and a refractory period that lasts several seconds (Slovin et al., 2002; Derdikman et al., 2003; Eytan and Marom, 2006; Gullo et al., 2010; Weihberger et al., 2013). With these properties in mind we adhere to a previously offered terminology and use the name Network Spike (NS) to designate evoked transient synchronous activity (Eytan and Marom, 2006; Shew et al., 2009). Trial-to-trial variations in the occurrence and the fine structure of stimulus evoked NSs were reported in both anesthetized and behaving animals as well as in reduced *ex-vivo* preparations (Vogels et al., 1989; Snowden et al., 1992; Arieli et al., 1996; Shahaf et al., 2008; Weihberger et al., 2013). The response variations reflect a multitude of factors that determine the network excitability status at the time of stimulus arrival. These include neuronal and synaptic noise, refractoriness of neuronal and synaptic activities and the context of ongoing activity within which a stimulus is applied (Arieli et al., 1996; Fox et al., 2006; Faisal et al., 2008; Weihberger et al., 2013).

Here we took advantage of a relatively controlled experimental approach to large-scale cortical networks developing *ex-vivo*, in order to expose the nature of response variations under repeated input over an extended range of time scales (from milliseconds to many minutes). We provide indications for the existence of a hierarchy of timescales in the structure of trial-to-trial response variations, ranging from sub-seconds to many minutes. We then show that response dynamics over minutes are reducible to the interplay of two instantaneous (i.e., single NS, sub-second scale) observables: (1) the *latency* from stimulus to the peak of the NS firing rate envelop, and (2) the *decay* duration from that peak to baseline activity. We show that these two instantaneous measures of the network excitability state—latency and decay duration—are differentially sensitive to specific pharmacological blockers of inhibitory and excitatory synaptic transmissions, suggesting that long-term network response variations reflect a dynamic excitation-inhibition ratio.

## 2. Materials and methods

### 2.1. Cell preparation

Cortical neurons were obtained from newborn rats (Sprague-Dawley) within 24 h after birth using mechanical and enzymatic procedures described in earlier studies (Marom and Shahaf, 2002). The neurons were isolated and plated directly onto substrate-integrated multi electrode arrays. They were allowed to develop into functionally and structurally mature networks over a period of 2 weeks and were used in experiments within the period of 2–6 weeks post plating. The number of plated neurons was in the order of 450,000, covering an area of about 380 mm^2^ with heat-inactivated horse serum (5%), glutamine (0.5 mM), glucose (20 mM), and gentamycin (10 μg/ml), and maintained in an atmosphere of 37°C, 5% CO_2_ and 95% air in an incubator as well as during the recording phases. An array of 60 Ti/Au extracellular electrodes, 30 μm in diameter, spaced 500 μm from each other (MultiChannelSystems, Reutlingen, Germany) was used. The insulation layer (silicon nitride) is pretreated with polyethyleneimine (Sigma, 0.01% in 0.1 M Borate buffer solution).

### 2.2. Electrophysiology

A commercial amplifier (MEA-1060-inv-BC, MCS, Reutlingen, Germany) with frequency limits of 150–3,000 Hz and a gain of × 1024 was used for obtaining data. Data was digitized using an acquisition board (PD2-MF-64-3M/12H, UEI, Walpole, MA, USA). Each channel was sampled at a frequency of 16 kHz, and detects electrical activity that might be originated from several sources (typically 2–3 neurons) as the recording electrodes were surrounded by several cell bodies. We have used a Simulink-based software for on-line control of data collection (see Zrenner et al., 2010 for more details). Voltage stimulation was applied in the form of a mono-phasic square pulse 200 μs 800–950 mV through extracellular electrodes using a dedicated stimulus generator (MCS, Reutlingen, Germany). Action potentials timestamps were detected on-line by threshold crossing of negative voltage. Detection of NSs was performed off-line using a previously described algorithm (Eytan and Marom, 2006) based on threshold crossing of the network firing rate (binned to 3 ms).

### 2.3. Pharmacology

Inhibitory synaptic transmission was blocked with Bicuculline-Methiodide (Sigma-Aldrich) that was incrementally added to the bathing solution (final concentrations used: 0.5, 1, 1.5, 2, 2.5, 3, 4, 5, and 7 μM). Excitatory synaptic blocker (APV; amino-5-phosphonovaleric, Sigma-Aldrich) was added to networks that are already under a Bicuculine blockade. Specifically, APV was added to networks that respond to stimuli in (or close to) a 1:1 manner, and where further application of Bicuculline did not change their response probability (5–12 μM Bicuculline). Final APV concentrations used were 50, 150, and 300 μM. In all the experiments with pharmacological manipulations the stimulation rate was chosen to be 1/5 s^−1^ or slower, thus maintaining high response probabilities; once tuned, the stimulation rate was kept fixed for each network throughout the experiment.

### 2.4. Data analysis

#### 2.4.1. Firing rate histograms and responsiveness

Once a NS was detected within 1 s following a stimulus, action potentials recorded in all the electrodes within 1500 ms following the stimulus were extracted. Post-stimulus time histograms were constructed using a 1 ms time bin, and smoothed with a 5 ms moving average. The *latency* measure was defined as the time between stimulus onset and the first maximum of the smoothed firing rate histogram. To compute the *decay* duration, the firing rate histogram was further smoothed by a wider window of 30–100 ms, thus avoiding impacts of oscillations within that phase on the measure. Decay duration was defined as the time between the histogram first maximum and the first drop below 0.15 spikes/ms along the falling phase of the NS.

#### 2.4.2. Fano factor

Network activity was represented as a point process (NS trains, composed of absolute detection times, i.e., not referenced to stimulation times). The variance of the network activity under repeating stimulation over extended time periods (24 h) was estimated by calculating its Fano factor (Scharf et al., 1995; Lowen and Teich, 1996). Count sequences Z(T(n)) were calculated using logarithmically spaced bin sizes T(n). For each count sequence the Fano factor, FF(T), defined as the variance of Z(T) divided by the mean of Z(T), was plotted as a function of the bin size T. The same analysis was applied to a surrogate data set generated by randomizing the inter-NS-intervals. The final rising section of the FF curves was fitted to a power law (α*T*^β^).

#### 2.4.3. Autocorrelation

The time series of latency and decay duration were used to calculate the autocorrelation of these processes, as if they were equally spaced in time, resulting in a warped time axis. Therefore, the autocorrelation was computed for “serial index lags” (see **Figure 5A**), instead of actual time lags, according to $\sum_{i\;=\;1}^{n-l}\frac{\left(x_{i}-\hat{\mu })(x_{l+i}-\hat{\mu }\right)}{{\sum_{i\;=\;1}^{n}\left(x_{i}-\hat{\mu }\right)}^{2}}$ Where the lag *l* is the difference between the serial indexes, in the stream of successful responses.

## 3. Results

The basic experimental procedure involved recording of activity while repetitively stimulating the network from a single site with constant amplitude at a constant rate. The data reported here originate from experiments (*n* = 27 networks) in which the stimulation amplitude was between 800 and 950 millivolts, delivered at a constant rate in the range of 1/3–1/12 s^−1^ (in different preparations and/or experimental sessions). The data were reduced to a series of network spike time-stamps using a threshold-based criterion for identification of synchronous activity (evoked as well as spontaneously occurring; see Section 2).

**Figure 1:**
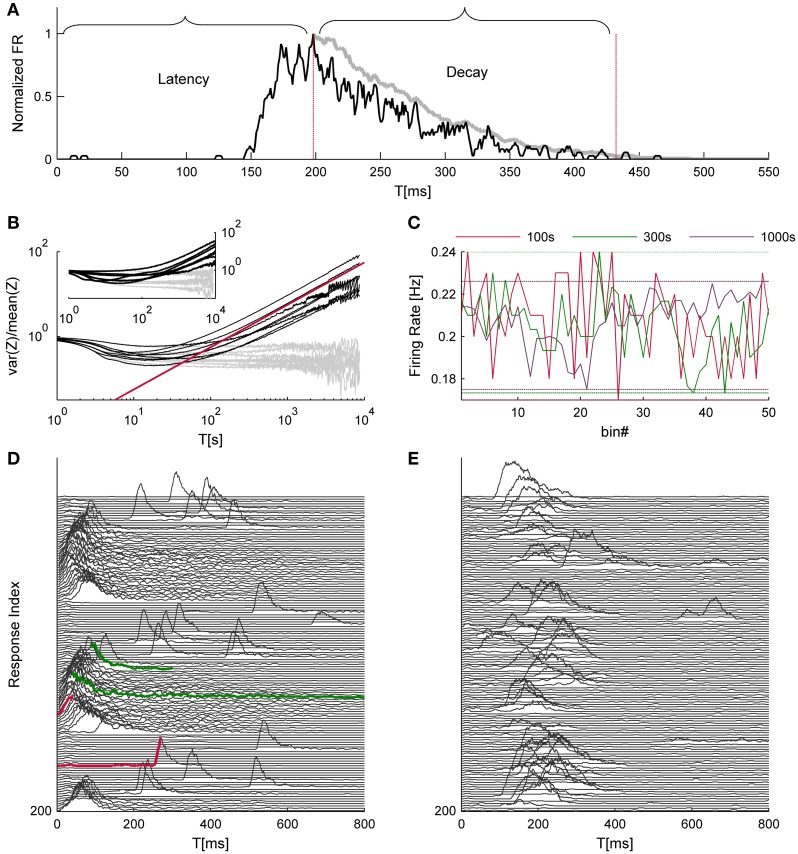
**Time hierarchy of network activity. (A)** shows a typical response to a single trial. The black trace depicts the network firing rate, binned at 1 ms and smoothed by a 5 ms moving average; the gray trace is a further smoothed version (30 ms moving average, in this case) of the falling phase of the NS, used to evaluate the *decay duration*. Both traces are normalized to their maximal firing rate. **(B)** depicts the variance over mean (i.e., Fano factor) of NSs count (*Z*) as a function of the integration time bins (*T*); data were collected from 8 networks that were continuously stimulated over 24 h at 1/5 s^−1^. The Gray lines depict identical analysis of the surrogate data set. The red line designates a power law with a unity exponent for reference purposes. The inset of **(B)** shows the same analysis for the cases of spontaneously evoked NSs (seven networks, 24 h time series). An intuitive example for temporal structures in network activity, which may give rise to such scale-free variations, is shown in **(C)** the range of fluctuations in the NS firing rate under repeating stimuli is comparable when calculated over 100, 300, and 1000 s bins (data is taken from one of the networks used for **(B)**. **(D,E)** demonstrate consecutive network responses, displaying two examples from a spectrum of observed response modes (every third response was removed to enhance visual clarity). Traces colored in red demonstrate instances of short vs. long response latencies; traces colored in green demonstrate instances of short vs. long decay durations.

The wide range of time scales present in neural population activity under stimulation is shown in Figure 1A demonstrates a typical evoked network spike; the latency from stimulus to the peak of the network spike, as well as the decay of activity from that peak, are processes that take place within the tens and hundreds of milliseconds, constituting the shortest timescales in our neuronal population data. The next timescale in the hierarchy is the network spike refractory period, which is the minimal time between two consecutive network spikes, ranging between 1 and 10 s (Robinson et al., 1993; Maeda et al., 1995; Eytan and Marom, 2006). The network refractory period is thus more than one order of magnitude longer compared to the above mentioned characteristic latency and decay times.

To identify further, slower timescales in the dynamics of network spikes under ongoing stimulation at a constant rate, we resorted to a measure of statistical variance that was introduced by Teich and colleagues for the analysis of spike number distributions (Teich, 1992). Specifically, time series of network spike occurrence were sliced to bins of *T* seconds durations, and the variance-to-mean ratio of event counts, a.k.a. Fano factor, was calculated as a function of *T*. Figure 1B shows the results of this analysis; the data originates from eight different networks (black lines) that were stimulated for 24 h at a rate of 1/5 s^−1^. The gray lines depict results of the same analytic procedure, applied to shuffled, surrogate data sets. At the very high temporal resolutions the Fano factor goes to unity, reflecting the Poisson nature of probability to capture an event within short time bins; this segment has no physiological significance. As *T* approaches the timescale of 1 s, the Fano factor starts declining, as the uniquely defined refractory period introduces regularity into the time series. (It is acknowledged that the stimulation cycle time is also a determinant at this temporal scale, but see below described analysis of spontaneous activity.) Should refractoriness be the sole source of temporal complexity, one would expect that the Fano factor will approach zero as *T* further increases, beyond the seconds scale. Apparently this is not the case: as the temporal resolution further decreases (i.e., larger *T*), the Fano factor steadily increases. The slopes at the right hand straight segments of the Fano factor curves (lower temporal resolutions) follow a power-law with an averaged exponent value of 0.64 ± 0.14, as if increasingly longer temporal structures are revealed by integrating the activity over longer temporal scales. In order to rule out the possibility that the increase of variability is a result of a drift in network responsiveness, we compared the response probability during the first hour and the last hour of the data and found no difference (0.22 ± 0.096 and 0.213 ± 0.075, respectively). The inset of Figure 1B demonstrates a similar phenomenon in the spontaneous activity recorded form 7 other networks over 24 h; the average exponent value here was 0.89 ± 0.0.21. Figures 1D,E recorded from two different networks, demonstrate different patterns of responsiveness, instances selected from a wide spectrum of possible temporal structures: one having a rather cyclic nature (D) and the other is more irregular (E); in these examples the responsiveness patterns of networks were stable throughout 50 min of recordings.

**Figure 2:**
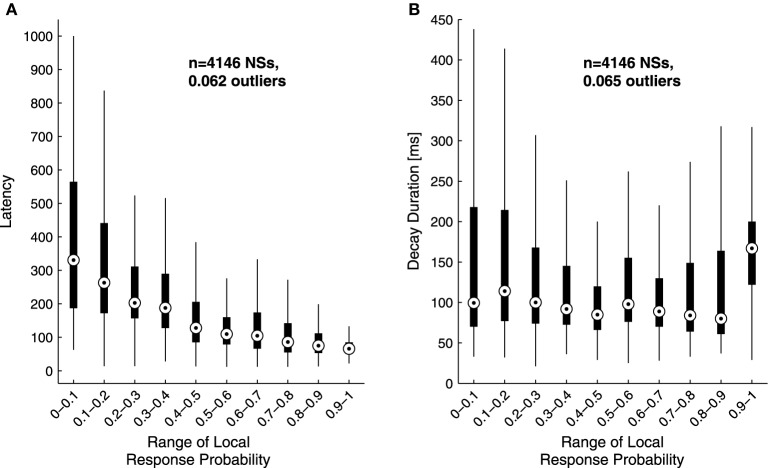
**Sensitivity of response latency (A) and decay (B) to response probability**. Latency **(A)** and decay duration **(B)** values of single responses were grouped according to their local response probability range in a data set of 4146 responses pooled from 17 networks stimulated at 1/3–1/5 s^−1^ over 50 min. The upper and lower edges of each box mark the 75 and 25 percentiles of the data; the circle within the box represents the group's median. Whiskers are extended up to an equivalent of ±2.7 standard deviations of each group;the fraction of outliers—i.e., falling outside the whiskers—is indicated in the plot. Note the inverse relation between response probability and latency.

**Figure 3:**
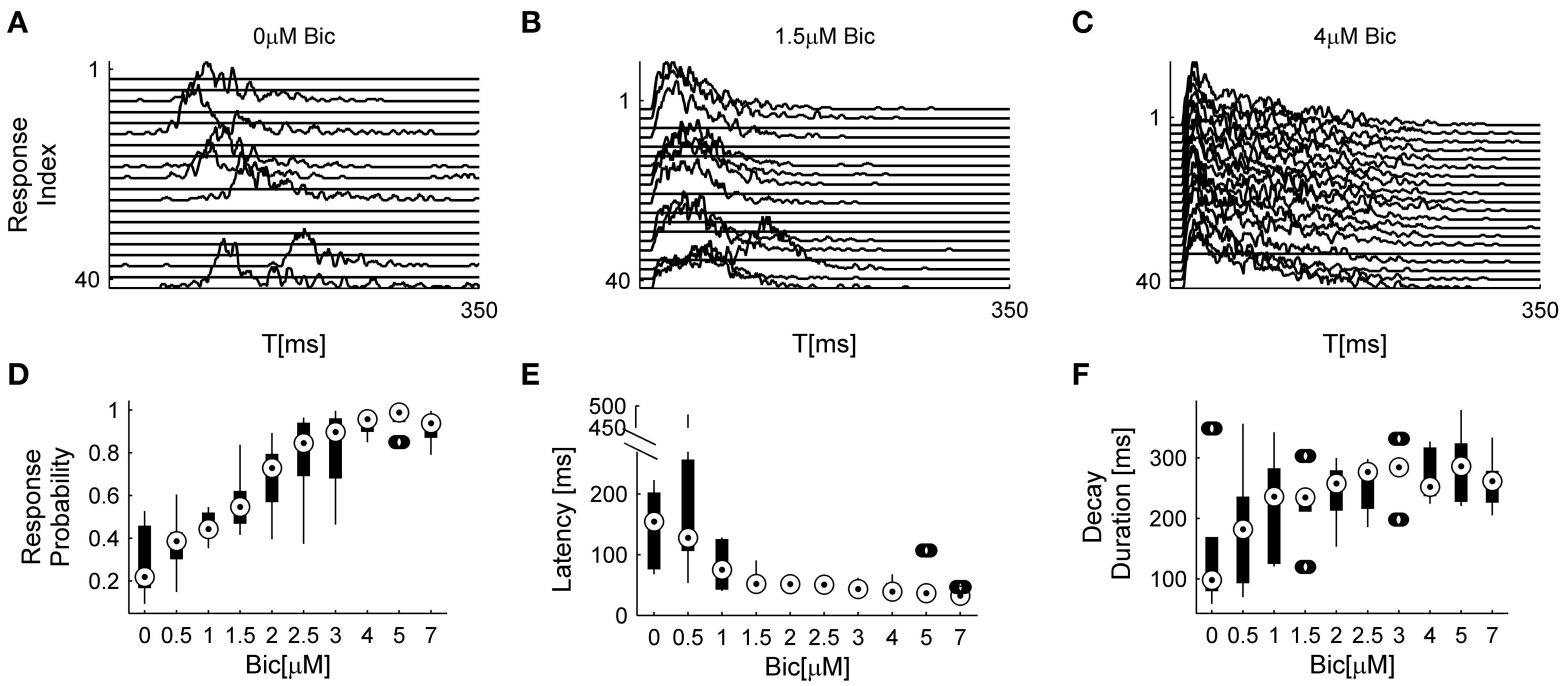
**Effects of inhibitory transmission on network responsiveness**. In **(A–C)** consecutive network responses to repeating stimulation (1/6 s^−1^) at different Bicuculline doses from a single network are shown (to enhance visual clarity every other response was omitted). **(D–F)** summarize the sensitivities of response probability, response latency, and response decay duration to Bicuculine (six networks). Box-plots represent the pooled distributions of local response probability **(D)** and response latency **(E)** collected from all six networks, each presented with 40 stimuli at ten increasingly applied doses. Out of 2400 responses, there are 0.0167 outliers of local response probability, 0.033 outliers of latency and 0.083 outliers of decay duration, designated by the circles outside the whiskers.

The analyses described above suggest that the temporal structure of series of network spikes under repeated stimuli markedly deviates from an independent and identically distributed process. While the limit of refractoriness is reasonably understood and well documented in the literature (Robinson et al., 1993; Maeda et al., 1995; Weihberger et al., 2013), the origin of correlations between evoked responses beyond the few seconds scale is poorly understood and, to the best of our knowledge, not described. In what follows we focus on response dynamics within the range of 10–1000 s. Many independent sources might contribute to the above dynamics of network responses over minutes. The data shown in Figures 1A–E suggest that these multiple sources may be reflected in immediate measurable response features. We chose to focus on the probability to evoke a network spike, as well as features of the network spike rise and falling phases, which—as shown below—seem related to each other. These relations are explored, at the level of pooled data, in Figure 2. For each evoked network spike we calculated the fraction of responses to stimuli in the vicinity of that network spike through a symmetric window of 10 preceding and 10 following stimulation events. Response latency and decay were defined as explained in Methods (see Figure 1A). Figure 2A shows a strong relation between response probability and latency of individual responses, in data pooled from 17 networks. The relation between response probability and decay duration is less obvious in the pooled data set. Previously reported studies show that the above features—response probability, latency and decay—as expressed in spontaneous activity (rate of occurrence, rise and falling phases of NSs, respectively), are sensitive to blockers of inhibitory and excitatory synaptic transmission (Maeda et al., 1995; Eytan and Marom, 2006; Gullo et al., 2010). We therefor studied the relations between bath concentration of synaptic blockers and the above features of the evoked responses. We gradually increased the concentration of Bicuculline (an antagonist of GABA_*A*_ receptors) from 0.5 μM up to 7 μM, and in each concentration we exposed the network to a series of 40 stimuli delivered at a low constant rate (within the range of 1/5–1/12 s^−1^). Figure 3 summarizes the results of these experiments (six networks). Overall, application of Bicuculline resulted in increased network response probability (Figure 3D), and decreased latency (Figure 3E). In addition—as may be seen in Figure 3F—gradual block of inhibitory synapses results in increased decay duration. Networks displaying baseline activities marked by high response probability and short latencies were not significantly affected by Bicuculline application (data not shown).

**Figure 4:**
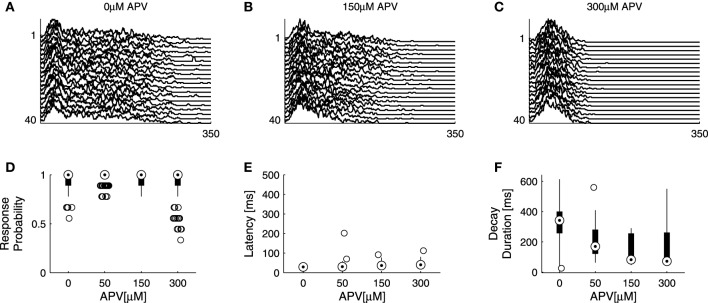
**Effects of excitatory transmission on network responsiveness**. APV is added on top of Bicuculine blockade (see Section 2), inducing an extreme case of effectively excitatory networks. **(A–C)** show consecutive network responses to slow (>1/7 s^−1^) repeating stimulation at different APV doses from a single network; to enhance visual clarity every other response was omitted. Sensitivities of response probability, latency and decay duration to APV are summarized in **(D–F)** using box-plots representing the pooled distributions of response probability **(D)**, latency **(E)** and decay duration **(F)** collected from four networks, each presented with 40 stimuli at four increasing doses. Out of 640 responses, there are 0.081 outliers of response probability, 0.00625 outliers of latency, and 0.0031 outliers of decay duration.

It is taken for granted that the coupling between network excitatory and inhibitory activities is such that, based on the results of Figure 3 no claim may be made regarding exclusive effects of Bicuculline on inhibition, as application of the drug may indirectly result in enhanced excitation. To expose—even if partially—the contribution of excitatory resources to network responsiveness, we applied increasing concentrations of APV, an NMDA antagonist, in the presence of high concentration (5 μM up to 12 μM) of Bicuculline. Under these conditions the responsiveness of the network is effectively determined by excitatory synaptic transmission. Admittedly, alternative blockers of excitatory transmission could have been used here; however, as we were interested in slow dynamics—the time course of network spike evolution, as well as long range correlations within series of responses, we chose to block the slow excitatory synaptic transmission. Figure 4 summarizes the effect of this experimental procedure (four networks). Within the applied range of APV dosage (50–300 μM), response probability remained more or less stable and the response latency was practically unaltered; but the network spike decay duration became significantly shorter. As might be expected, considering the network spike as an excitable event of the network, higher doses of APV (>300 μM) induced a decrease of response probability accompanied by an increase of response latency (data not shown). The overall picture emerging from the experiments of Figures 3, 4 is that response latency is more sensitive to the availability of inhibition, whereas decay duration shows greater sensitivity to the availability of excitation, at least when estimated at relatively disinhibited regimes. Of course, other features of the NS were also affected by application of these synaptic blockers; for instance, NS amplitude increases under bicuculline, but is not systematically affected by APV. We focus on latency and decay.

**Figure 5:**
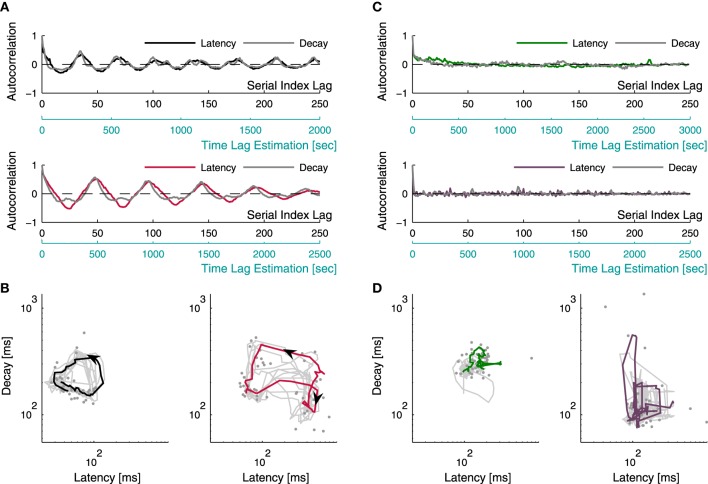
**Latency-decay relations**. Latency and decay duration were estimated from a data set of 17 networks during 50 min of repeating stimulation at 1/3–1/5 s^−1^. Examples for the different manners by which latency and decay duration behave and interact are presented. **(A)** depicts the autocorrelations of the time series of latency (black/red traces) and decay duration (gray traces) from two different networks, recorded over 50 min; both manifesting oscillations persisting over minutes. The autocorrelations are displayed with two lag-axes: the upper axis (black) displays gaps between serial numbers of consecutive successful responses (see Section 2), while the lower axis (light blue) displays an estimation of the actual time lags, evaluated from the average inter-response-intervals. Pairs of latency and decay duration of individual responses, obtained from the two networks of **(A)** (accordingly colored), are plotted using a log-log scale in **(B)**, demonstrating organized activity of repeating trajectories on the latency-decay plane. The gray dots represent individual responses from a single trajectory, the colored curves depict the smoothed version of this trajectory (calculated using a moving average procedure), and the gray curves depict 50 min of the smoothed traces of latency-decay pairs from each network. **(C)** shows the autocorrelation of latency and decay duration time series from two other networks demonstrating the more common cases, where networks vagrantly explore the latency-decay plane with no apparent repeating trajectories. The latency series autocorrelations are depicted by the colored lines, and the decay series autocorrelations are depicted in gray. The top inset displays a case of long lasting correlations, while the bottom inset demonstrates a case of no correlation at all in both measures. The latency-decay traces of these networks are displayed in **(D)**, the colored lines demonstrate an apparently disorganized wandering across the latency-decay plane within a trace of 50 consecutive responses.

Let us assume, based on the above results, that latency and decay duration represent aspects of inhibition and excitation. With this assumption in mind one might consider the use of the relations between these two measures of the individual response, as means to explore the stability (or, instability) of network excitation-inhibition ratio. The long term dynamics of both measures—latency and decay—as well as the interaction between them, may be intuited from the four examples shown in Figure 5. These four examples (pulled out from a data set of 17 networks) represent the two extremes of a spectrum revealed by repeatedly stimulating networks at 1/3–1/5 Hz over 50 min; this spectrum extends from narrow confined dynamics, through seemingly capricious, to well-organized several minutes-long trajectories across the latency-decay plane. Figure 5A shows the autocorrelation function of latency and decay series from two networks exhibiting oscillating autocorrelation of both measures, over minutes. Figure 5B represents latency-decay pairs obtained from individual responses of these two networks, forming well organized trajectories that persist many minutes. Although the autocorrelations of latency and decay in Figure 5A are highly coordinated, their ratio is not at all fixed, as may be inferred from the trajectories of Figure 5B. Rather, the ratio of latency to decay covers a wide dynamic range in their joint plane, markedly deviates from the main diagonal. (Note: the top panel of Figure 5A is calculated from the same network used to produce the population firing rate traces in Figure 1C). The dynamics shown in Figures 5A,B represent a case that is extreme: these well-formed dynamics occupy relatively short segments of the entire data set. Most of the networks studied here did not exhibit coherent trajectories that last as long. Rather, their dynamics ranged between long lasting correlations (hundreds of seconds, Figure 5C top inset) to no correlation at all (Figure 5C bottom inset). Figure 5D demonstrates the latency-decay joint distribution from these networks and their vagrant responsiveness pattern; the colored lines depict segments of 50 consecutive responses, comparable with the colored trajectory-segments lengths presented in Figure 5B. The existence of long lasting correlations does not seem to be related to the spread of latency and decay duration of a given network. It might, however, be related to a higher center of mass along the decay axis. Overall, to the extent that latency and decay duration are proxies of inhibition and excitation, the data of Figure 5 implies that E/I should be treated as a dynamic variable over long temporal scales, rather than a parameter.

Note that the dependency of the Fano factor on T (Figure 1B) is consistent for all networks (which would suggest the same dynamics for network excitability), yet the dynamics in the latency-decay plane and the autocorrelations structure are very diverse. This gap can be bridged by the fact that the data used in Figure 5 consist of relatively short recording segments (50 min), while the analyses shown in Figure 1B is calculated over long recordings (24 h). Figure 1C demonstrates in a more intuitive manner how such high order statistics analysis may yield similar results over a diverse dataset. The power law growth of the FF with a growing observation window suggests a scale-free variance of activity; indeed, when the firing rate of the network is calculated over different time bins, the fluctuations on different timescales spans comparable ranges.

## 4. Discussion

We provide experimental indications linking slow variations in network responsiveness over the scale of minutes, to sub-second features of individual responses, and point to a potential connection between these variations and a dynamical excitation-inhibition ratio. The sequence of our arguments begins with a description of correlations in time series of stimulus-evoked NSs occurrence, beyond the few seconds scale. We show that network responsiveness is inversely correlated with response latency. Aided by pharmacological manipulations, we point to differential sensitivity of the NS's latency and decay to excitatory and inhibitory synaptic transmission. We then reduce the multidimensional data of long-term population response variability to instantaneous changes in these two readily observable measures. Hence, we offer a method to instantaneously estimate the network state in terms of its E/I ratio directly at the population level. While not free of limitations, this estimation of E/I ratio is less sensitive to synaptic filtering introduced to estimations that are based on intracellular measurements. We demonstrate a spectrum of manners at which latency and decay duration behave and interact. To the extent that latency and decay reflect aspects of inhibitory and excitatory network activity, our observation suggests a view on the excitation-inhibition ratio being a dynamical variable over extended time scales; an interpretation discussed in what follows.

Excitation-inhibition ratio is a determinant of network activity signature (Brunel, 2000). It is often assumed—based on theoretical considerations—that this ratio is balanced (Shadlen and Newsome, 1994; van Vreeswijk and Sompolinsky, 1996; Shadlen and Newsome, 1998; Vogels et al., 2005). Experimental efforts to validate the balanced excitation-inhibition assumption are not conclusive: alongside supportive observations (Haider et al., 2006; Okun and Lampl, 2008; Xue et al., 2014), the balanced E/I assumption was challenged by others (Stevens and Zador, 1998; Wehr and Zador, 2003; Heiss et al., 2008). The observations reported in the present study do not contradict the abstract notion of *effective* excitation and inhibition being somehow matched such that networks remain globally responsive over time. Rather, we point to the statistical nature of fluctuations in the ratio between effective exciting and inhibiting forces, as reflected in features of network responsiveness. These results may justify opening a discussion on the mechanism by which the effective ratio is self-organized, a mechanism that gives rise to broadly distributed fluctuations. The framework of self-organized criticality is one possible path to take (Plenz and Schuster, 2014); but many other paths toward self-organization are plausible, paths that involve machineries at multiple spatial and temporal scales.

We imagine at least three challenges that are entailed by the above notion of excitation-inhibition ratio as a dynamical system variable, rather than a parameter. The *first* challenge concerns modeling. The level of organization of the observed phenomenon (variability of population response) precludes models that focus on microscopic, this-or-that channel or synaptic receptor mechanisms underlying the rich network dynamics. In fact, even the pharmacological manipulations we have applied are limited relative to the range of synaptic transmission mechanisms involved in propagation in these networks; for instance, much of the activity is propagated through AMPA receptors, which we have not blocked. It might be more appropriate to use the terms “exciting forces' and “restoring forces” rather than “excitation” and “inhibition,” thus shifting the load from local synaptic processes toward a richer repertoire of potential mechanisms that contribute to changes in network dynamics. Models that are formulated in terms of global adaptation of both exciting and restoring forces and their interaction with time scales of stimulation and spontaneous activity are wanted. Such models might shed light on possible connections between the dynamics of stimulus evoked responses reported here and the well-documented complex statistics of spontaneous series of network spikes (Segev et al., 2002; Beggs and Plenz, 2003; Wagenaar et al., 2006; Mazzoni et al., 2007). The *second* challenge we envision is related to functional aspects of network activity in general, and the impacts of slow dynamics of exciting and restoring forces on the efficacy of different representation schemes, in particular. Whether such schemes involve population or spike-time “neural codes,” their sensitivity to slow network dynamics should be considered. One might imagine scenarios where slow network dynamics of the kind described here can have constructive impacts on the ability of neural systems to explore and adapt to a changing environment. The *third* challenge is even more general; it concerns the inherent tension between approaches that heavily rely on structural measures and approaches that are more concerned with “effective” measures in providing insightful information on neural systems dynamics and function. The multiple timescales of network responsiveness as well as the dynamic exciting-restoring forces, emerge from an allegedly stable structure, at least in terms of the number of excitatory and inhibitory neurons and the number of their synapses. Indeed, it is difficult to imagine the dynamics over minutes as presented here being determined by ongoing changes in structural network parameters. In this respect our results further contribute to recent calls that challenge attempts to relate the structure of neural networks to their dynamics (Marder et al., 2014).

Whether or not our interpretations that are based on *in-vitro* experimental analyses may be generalized, the very possibility of network-level exciting and restoring forces being dynamical variables seems to deserve serious consideration by those interested in theoretical and applied aspects of neural response variation.

### Conflict of interest statement

The authors declare that the research was conducted in the absence of any commercial or financial relationships that could be construed as a potential conflict of interest.

## References

[B1] Arieli A. Sterkin A. Grinvald A. Aertsen A. (1996). Dynamics of ongoing activity: explanation of the large variability in evoked cortical responses. Science 273, 1868–1871. 10.1126/science.273.5283.1868 8791593

[B2] Beggs J. M. Plenz D. (2003). Neuronal avalanches in neocortical circuits. J. Neurosci. 23, 11167–11177. 14657176 10.1523/JNEUROSCI.23-35-11167.2003 PMC6741045

[B3] Brunel N. (2000). Dynamics of sparsely connected networks of excitatory and inhibitory spiking neurons. J. Comput. Neurosci. 8, 183–208. 10.1023/A:1008925309027 10809012

[B4] Derdikman D. Hildesheim R. Ahissar E. Arieli A. Grinvald A. (2003). Imaging spatiotemporal dynamics of surround inhibition in the barrels somatosensory cortex. J. Neurosci. 23, 3100–3105. 12716915 10.1523/JNEUROSCI.23-08-03100.2003 PMC6742298

[B5] Eytan D. Marom S. (2006). Dynamics and effective topology underlying synchronization in networks of cortical neurons. J. Neurosci. 26, 8465–8476. 10.1523/JNEUROSCI.1627-06.2006 16914671 PMC6674346

[B6] Faisal A. Selen L. Wolpert D. (2008). Noise in the nervous system. Nat. Rev. Neurosci. 9, 292–303. 10.1038/nrn2258 18319728 PMC2631351

[B7] Fox M. D. Snyder A. Z. Zacks J. M. Raichle M. E. (2006). Coherent spontaneous activity accounts for trial-to-trial variability in human evoked brain responses. Nat. Neurosci. 9, 23–25. 10.1038/nn1616 16341210

[B8] Gullo F. Mazzetti S. Maffezzoli A. Dossi E. Lecchi M. Amadeo A.. (2010). Orchestration of “presto” and “largo” synchrony in up-down activity of cortical networks. Front. Neural Circuits 4:11. 10.3389/fncir.2010.00011 20461235 PMC2866559

[B9] Haider B. Duque A. Hasenstaub A. R. McCormick D. A. (2006). Neocortical network activity *in vivo* is generated through a dynamic balance of excitation and inhibition. J. Neurosci. 26, 4535–4545. 10.1523/JNEUROSCI.5297-05.2006 16641233 PMC6674060

[B10] Heiss J. E. Katz Y. Ganmor E. Lampl I. (2008). Shift in the balance between excitation and inhibition during sensory adaptation of s1 neurons. J. Neurosci. 28, 13320–13330. 10.1523/JNEUROSCI.2646-08.2008 19052224 PMC6671605

[B11] Lowen S. Teich M. (1996). The periodogram and allan variance reveal fractal exponents greater than unity in auditory-nerve spike trains. J. Acoust. Soc. Am. 99, 3585–3591. 10.1121/1.414979 8655790

[B12] Maeda E. Robinson H. Kawana A. (1995). The mechanisms of generation and propagation of synchronized bursting in developing networks of cortical neurons. J. Neurosci. 15, 6834–6845. 7472441 10.1523/JNEUROSCI.15-10-06834.1995 PMC6578010

[B13] Marder E. O'Leary T. Shruti S. (2014). Neuromodulation of circuits with variable parameters: small circuits reveal principles of state-dependent and robust neuromodulation. Annu. Rev. Neurosci. 37, 329–346. 10.1146/annurev-neuro-071013-013958 25032499

[B14] Marom S. Shahaf G. (2002). Development, learning and memory in large random networks of cortical neurons: lessons beyond anatomy. Q. Rev. Biophys. 35, 63–87. 10.1017/S0033583501003742 11997981

[B15] Mazzoni A. Broccard F. D. Garcia-Perez E. Bonifazi P. Ruaro M. E. Torre V. (2007). On the dynamics of the spontaneous activity in neuronal networks. PLoS ONE 2:e439. 10.1371/journal.pone.0000439 17502919 PMC1857824

[B16] Okun M. Lampl I. (2008). Instantaneous correlation of excitation and inhibition during ongoing and sensory-evoked activities. Nat. Neurosci. 11, 535–537. 10.1038/nn.2105 18376400

[B17] Plenz D. Schuster H. G. (2014). Criticality in Neural Systems. New York, NY: Wiley-VCH.

[B18] Robinson H. Kawahara M. Jimbo Y. Torimitsu K. Kuroda Y. Kawana A. (1993). Periodic synchronized bursting and intracellular calcium transients elicited by low magnesium in cultured cortical neurons. J. Neurophysiol. 70, 1606–1616. 8283217 10.1152/jn.1993.70.4.1606

[B19] Scharf R. Meesmann M. Boese J. Chialvo D. R. Kniffki K.-D. (1995). General relation between variance-time curve and power spectral density for point processes exhibiting 1/f β-fluctuations, with special reference to heart rate variability. Biol. Cybern. 73, 255–263. 10.1007/BF00201427 7548313

[B20] Segev R. Benveniste M. Hulata E. Cohen N. Palevski A. Kapon E.. (2002). Long term behavior of lithographically prepared *in vitro* neuronal networks. Phys. Rev. Lett. 88:118102. 10.1103/PhysRevLett.88.118102 11909430

[B21] Shadlen M. Newsome W. (1994). Noise, neural codes and cortical organization. Curr. Opin. Neurobiol. 4, 569–579. 10.1016/0959-4388(94)90059-0 7812147

[B22] Shadlen M. N. Newsome W. T. (1998). The variable discharge of cortical neurons: implications for connectivity, computation, and information coding. J. Neurosci. 18, 3870–3896. 9570816 10.1523/JNEUROSCI.18-10-03870.1998 PMC6793166

[B23] Shahaf G. Eytan D. Gal A. Kermany E. Lyakhov V. Zrenner C.. (2008). Order-based representation in random networks of cortical neurons. PLoS Comput. Biol. 4:e1000228. 10.1371/journal.pcbi.1000228 19023409 PMC2580731

[B24] Shew W. L. Yang H. Petermann T. Roy R. Plenz D. (2009). Neuronal avalanches imply maximum dynamic range in cortical networks at criticality. J. Neurosci. 29, 15595–15600. 10.1523/JNEUROSCI.3864-09.2009 20007483 PMC3862241

[B25] Slovin H. Arieli A. Hildesheim R. Grinvald A. (2002). Long-term voltage-sensitive dye imaging reveals cortical dynamics in behaving monkeys. J. Neurophysiol. 88, 3421–3438. 10.1152/jn.00194.2002 12466458

[B26] Snowden R. J. Treue S. Andersen R. A. (1992). The response of neurons in areas v1 and mt of the alert rhesus monkey to moving random dot patterns. Exp. Brain Res. 88, 389–400. 10.1007/BF02259114 1577111

[B27] Stevens C. F. Zador A. M. (1998). Input synchrony and the irregular firing of cortical neurons. Nat. Neurosci. 1, 210–217. 10.1038/659 10195145

[B28] Teich M. C. (1992). Fractal Neuronal Firing Patterns. Boston: Academic.

[B29] Uhlhaas P. J. Pipa G. Lima B. Melloni L. Neuenschwander S. Nikolic D.. (2009). Neural synchrony in cortical networks: history, concept and current status. Front. Integr. Neurosci. 3:17. 10.3389/neuro.07.017.2009 19668703 PMC2723047

[B30] van Vreeswijk C. Sompolinsky H. (1996). Chaos in neuronal networks with balanced excitatory and inhibitory activity. Science 274, 1724–1726. 10.1126/science.274.5293.1724 8939866

[B31] Vogels R. Spileers W. Orban G. (1989). The response variability of striate cortical neurons in the behaving monkey. Exp. Brain Res. 77, 432–436. 10.1007/BF00275002 2792290

[B32] Vogels T. P. Rajan K. Abbott L. F. (2005). Neural network dynamics. Annu. Rev. Neurosci. 28, 357–376. 10.1146/annurev.neuro.28.061604.135637 16022600

[B33] Wagenaar D. A. Pine J. Potter S. M. (2006). An extremely rich repertoire of bursting patterns during the development of cortical cultures. BMC Neurosci. 7:11. 10.1186/1471-2202-7-11 16464257 PMC1420316

[B34] Wehr M. Zador A. M. (2003). Balanced inhibition underlies tuning and sharpens spike timing in auditory cortex. Nature 426, 442–446. 10.1038/nature02116 14647382

[B35] Weihberger O. Okujeni S. Mikkonen J. E. Egert U. (2013). Quantitative examination of stimulus-response relations in cortical networks *in vitro*. J. Neurophysiol. 109, 1764–1774. 10.1152/jn.00481.2012 23274313

[B36] Xue M. Atallah B. V. Scanziani M. (2014). Equalizing excitation-inhibition ratios across visual cortical neurons. Nature 511, 596–600. 10.1038/nature13321 25043046 PMC4117808

[B37] Zrenner C. Eytan D. Wallach A. Thier P. Marom S. (2010). A generic framework for real-time multi-channel neuronal signal analysis, telemetry control, and sub-millisecond latency feedback generation. Front. Neurosci. 4:173. 10.3389/fnins.2010.00173c 21060803 PMC2972682

